# Robust Inertial Measurement Unit-Based Attitude Determination Kalman Filter for Kinematically Constrained Links

**DOI:** 10.3390/s19040768

**Published:** 2019-02-13

**Authors:** Jung Keun Lee, Mi Jin Choi

**Affiliations:** Inertial Motion Capture Lab, Department of Mechanical Engineering, Hankyong National University, Anseong 17579, Korea; alwlsdong@gmail.com

**Keywords:** attitude, constraint, external acceleration, Kalman filter, kinematic inertial measurement unit

## Abstract

The external acceleration of a fast-moving body induces uncertainty in attitude determination based on inertial measurement unit (IMU) signals and thus, frequently degrades the determination accuracy. Although previous works adopt acceleration-compensating mechanisms to deal with this problem, they cannot completely eliminate the uncertainty as they are, inherently, approaches to an underdetermined problem. This paper presents a novel constraint-augmented Kalman filter (KF) that eliminates the acceleration-induced uncertainty for a robust IMU-based attitude determination when IMU is attached to a constrained link. Particularly, this research deals with an acceleration-level kinematic constraint derived on the basis of a ball joint. Experimental results demonstrate the superiority of the proposed constrained KF over the conventional unconstrained KF: The average accuracy improved by 1.88° with a maximum of 4.18°. More importantly, whereas the accuracy of conventional KF is dependent to some extent on test acceleration conditions, that of the proposed KF is independent of these conditions. Due to the robustness of the proposed KF, it may be applied when accurate attitude estimation is needed regardless of dynamic conditions.

## 1. Introduction

Attitude determination based on the inertial measurement unit (IMU) is widely used in various fields, such as human motion tracking, sports science, and robotics [1,2,3,4,5,6,7,8]. For example, the attitude or the orientation, instead of position information, can be effectively used to track limb movements using IMU alone [9,10] or by the fusing of IMU and aiding sensors [11,12]. A typical IMU consists of a triaxial accelerometer and a triaxial gyroscope, and Kalman filtering is the most popular framework for the attitude determination [13,14]. Despite the variety of detailed approaches to Kalman filters (KF), the basic concepts of IMU-based attitude KFs are all the same: The attitude is predicted by time-integrating the gyroscope signals, but the unbounded drift is caused by the error accumulation in the course of integration. For the correction, the accelerometer provides a drift-free vertical reference by sensing gravity [14,15].

However, the case where the accelerometer signal is dominated by the gravitational acceleration is limited to the static condition, as the accelerometer signal in dynamic conditions is the sum of the gravitational acceleration and the external acceleration [15]. In order to distinguish between the two, attitude information is needed. It should be remembered that the attitude is, nonetheless, what we want to determine. Therefore, IMU-based attitude determination under dynamic conditions is a type of underdetermined problem because the accelerometer signal used for the correction has two unknowns: The attitude and the external acceleration. Accordingly, the external acceleration of the moving body induces uncertainty in the attitude determination, and thus, frequently degrades the determination accuracy. For example, the attitude determination can be required in the motion analysis of athletes and fast-moving systems that are frequently exposed to considerable amounts of acceleration. Consequently, the acceleration-related uncertainty becomes a critical problem.

In order to deal with this problem, a number of attitude estimation algorithms adopt acceleration-compensating mechanisms. Such mechanisms include simple switching techniques (e.g., measurement vector switching [14] and measurement covariance matrix switching [5]) and acceleration model-based mechanisms using the Markov chain [16,17]. Despite these efforts however, previous approaches cannot completely eliminate the acceleration-related uncertainty as they are, inherently, approaches to an underdetermined problem. For this reason, the improvement of determination accuracy under dynamic conditions remains limited.

This study may provide a solution to the aforementioned problem. The key underlying concept of this research is that the attitude determination for an unconstrained body is an underdetermined problem, whereas the attitude determination for a constrained body does not have to be such. Hence, this paper presents a novel constraint-augmented KF that eliminates acceleration-related uncertainty for robust IMU-based attitude determination, when IMU is attached to a constrained link. Specifically, this research deals with a kinematic constraint generated by a ball-and-socket joint.

Over the last few years, several approaches have been developed to augment constraints within the KF framework. They include estimate projection [18], gain projection [19], and measurement augmentation [20]. In the field of IMU applications, kinematic constraints have been utilized to achieve considerably consistent attitude estimations, typically for human motion tracking [9,21,22,23,24,25,26,27]. In Reference [19], constraints are used to ensure that segments remain connected at the joints in their biomechanical model. Luinge et al. [9] and Zhang et al. [23] embedded the geometric constraint in the elbow joint (i.e., the adduction angle is restricted to a considerably small angle.), to a KF and a particle filter, respectively. In Reference [22], anatomical constraints, such as joint angle limits and limitations of limb motions, are used to transform the attitude-determination problem to an optimization problem (i.e., the estimated attitudes should optimize the consistency with accelerometer measurements and satisfy constraints at the same time). Note that in previous studies, the use of kinematic constraints for the attitude determination basically lies at the ‘position level’ by exploiting, for example, the connectivity of segments and the restriction of the range of motion, for human motion analysis. In Reference [28], a constraint equation based on the velocity of the elbow joint, relative to both the upper arm and the forearm, is applied for a better estimation of the forearm and the upper arm rotation. 

This study focuses on the ‘acceleration-level’ constraint for dynamic operating conditions, considering fast-moving athletes and various systems with abrupt motion changes. It should be noted that the acceleration-level kinematic constraint contains terms of the acceleration and angular velocity, which are related to signals of the accelerometer and the gyroscope. Therefore, the acceleration-level kinematic constraint can be augmented in a measurement model of a KF structure. By doing so, the aforementioned acceleration-related uncertainty can be eliminated, and thus, the attitude-determination problem evades being an underdetermined problem. In Reference [29], the acceleration-level constraint equation was used to determine segment-fixed vectors from IMUs to the center of the rotation of the joint, in the course of the joint angle estimation. However, so far the acceleration-level constraint has not been used for the attitude determination, by replacing the Markov chain-based acceleration model with the acceleration-level constraint.

In this study, the acceleration-level kinematic constraint, associated with a ball-and-socket joint, is augmented in the measurement model of a state-of-the-art attitude-determination KF, in order to determine the attitude without the acceleration-induced uncertainty. Although this paper provides a proof-of-concept with a ball-and-socket joint constraint as an example, the proposed approach may open up many directions for future research. Experimental results are provided to evaluate the performance of the proposed constrained KF in comparison with conventional unconstrained KFs under various accelerated conditions.

## 2. Methods

### 2.1. Problem Definition and Sensor Modeling

**Figure 1:**
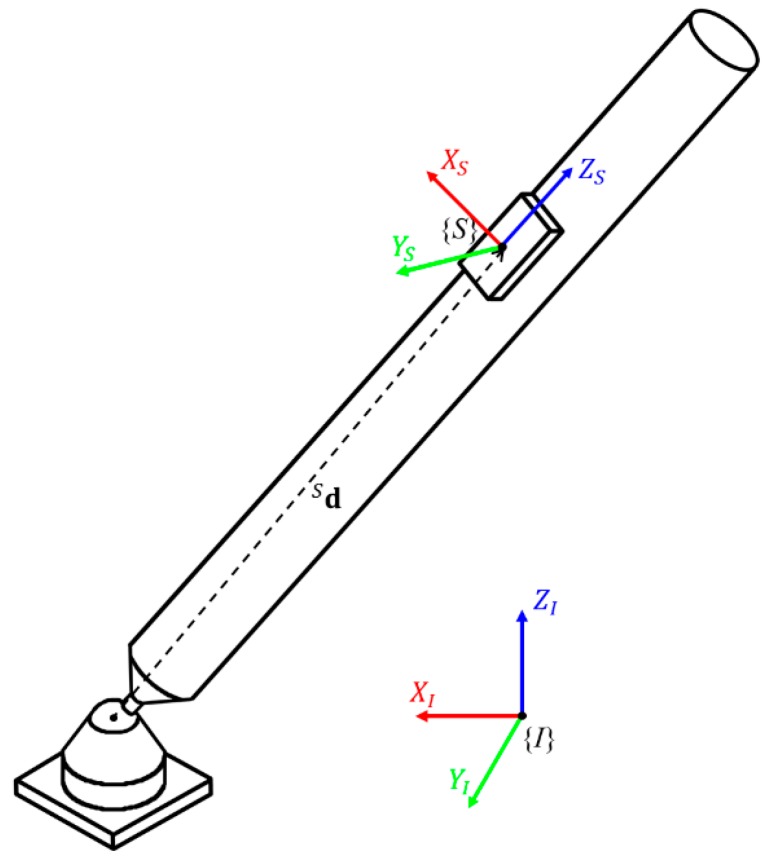
An inertial measurement unit (IMU) attached to a kinematically-constrained link by a ball-and-socket joint.

Let us assume that an IMU, to which the sensor-fixed frame *S* is attached, rotates with respect to a fixed inertial reference frame, *I* (see Figure 1). The rotation matrix RIS of {*I*}, with respect to {*S*}, can be interpreted as a set of three-unit axis column vectors of {*I*}, written in {*S*}. That is,

(1) RIS=[XSIYSIZSI]

Note that the last column, ZSI, represents the attitude, as it can be used to calculate roll and pitch, i.e., γ=atan2(ZS2,ZS3) and β=atan2(−ZS1,ZS2/sinγ), respectively, when ZSI=[ZS1ZS2ZS3]T. Hence, the purpose of the proposed KF is to determine ZSI. (henceforth, written simply as ZS, for convenience). In this paper, the axis vectors are represented by capital letters to distinguish them from other variables.

Sensor signals from the accelerometer (*A*) and gyroscope (*G*) are modeled, respectively, as follows:

(2) yA=gS+aS+nA

(3) yG=ωS+nG

where gS is the gravity vector; ω is the angular velocity; a is the external acceleration; and the n’s are the measurement noises that are assumed to be zero-mean white Gaussian and also uncorrelated each other [17]. It is natural that vectors in sensor signals are observed in the sensor-frame coordinates, as indicated by the left superscript, *S*.

When the Z-axis of the inertial frame points vertically upward (which is the most common setup), gS in Equation (2) can be expressed in terms of ZS, i.e., gS=gZS, where *g* = 9.8 m/s^2^. It should be noted that Equation (2) has two unknowns that are interdependent: ZS and aS.

### 2.2. Acceleration-Level Ball Joint Constraint

This study deals with a ball-and-socket joint (or simply, a ball joint) constraint, which is defined by the condition that the center of the ball coincides with the center of the socket. This condition only restricts three translational degrees of freedom of a point in the ball-side link, relative to the socket-side link, but allows three rotational degrees of freedom. Then, we assumed that an IMU is mounted on a link, which was connected to another link via a ball joint. When observed in the sensor frame, the vector from the joint center to the origin of the sensor frame, dS, was constant during motion and could be predetermined during the initial calibration procedure. Moreover, the acceleration of the sensor could be considered as the sum of the acceleration of the joint center and the acceleration caused by the rotation of that sensor around the joint center. 

As a simplified system, the socket-side link was firmly fixed to the ground in this study (see Figure 1). Accordingly, the joint center’s acceleration was zero, and the acceleration of the sensor was as follows:

(4) aS=([ω˙S×]+[ωS×][ωS×])dS,

which is a function of the angular velocity and acceleration, and the predetermined constant position vector. Here, $\dot{\omega }$ (having the overhead dot) represents the first-time derivative of ω, and [ω×] is a 3 × 3 skew symmetric matrix to represent the standard vector cross product. Because the ideal angular velocity ωS, in Equation (4) was unavailable in practice because of the measurement noise, it was necessary to express Equation (4) using the actual gyroscope output, $y_{G}$. Therefore, if we replace ωS with $y_{G}-n_{G}$, according to Equation (3), and use $\left[(y_{G}-n_{G})\times ]=[y_{G}\times ]-[n_{G}\times \right]$, the acceleration-level kinematic constraint Equation (4) becomes

(5) aS=([y˙G×]+[yG×][yG×])dS+aSε

where the error of the acceleration equation, aSε, is derived as

(6) aSε=[dS×]n˙G−([dS×][yG×]−2[yG×][dS×])nG

In Equation (5), ${\dot{y}}_{G}$ is obtained by numerical differentiation, i.e., ${\dot{y}}_{G,t}\approx (y_{G,t}-y_{G,t-1})/\Delta t$, where Δt is the time step size. Furthermore, for the derivation of Equation (5), $[y_{G}\times ][n_{G}\times ]-[n_{G}\times ][y_{G}\times ]=\left[([y_{G}\times ]n_{G})\times \right]$ is applied and $\left[n_{G}\times ][n_{G}\times \right]$ is ignored. Finally, by using the relationship [a×]b=−[b×]a, the gyroscope noise $n_{G}$ and its first-time derivative ${\dot{n}}_{G}$ are set at the right side of the corresponding terms to enable the formulation of the noise covariance matrix.

### 2.3. Constraint-Augmented Kalman Filter

The proposed algorithm was based on our previous work [17], which was an attitude-determination algorithm, using a linear KF. The basic structure of the proposed KF can be defined by the following process and measurement models:

(7) $$x_{t}=\Phi_{t-1}x_{t-1}+w_{t-1}$$

and

(8) $$z_{t}=H_{t}x_{t}+v_{t}$$

where is the state vector, z is measurement vector, Φ is the state transition matrix, H is the observation matrix, and w and v are the white Gaussian process and measurement noises, respectively. Because the purpose of our KF is to determine ZS, the state vector is simply defined as x=ZS.

First, the process model based on the strapdown integration of the gyroscope measurement is

(9) ZSt=(I−Δt[yG,t−1×])ZSt−1+Δt(−[ZSt−1×])nG

From Equation (7) and Equation (9), the transition matrix $\Phi_{t-1}$, and process noises $w_{t-1}$, are $I-\Delta t[y_{G,t-1}\times ]$ and Δt(−[ZSt−1×])nG, respectively. Moreover, the process noise covariance matrix $Q_{t-1}$, defined by $E\left[w_{t-1}w_{t-1}^{T}\right]$, is −Δt2[ZSt−1×]ΣG[ZSt−1×], where *E* is the expectation operator. The covariance matrix of the gyroscope’s measurement noise, $\Sigma_{G}$, is set equal to $\Sigma_{G}=\sigma_{G}^{2}I$, where $\sigma_{G}^{2}$ is the gyroscope noise variance. One may refer to Reference [17] for details of the process model.

Second, the measurement model was based on the accelerometer signal, Equation (2), combined with the acceleration-level kinematic constraint, Equation (5), derived in Section 2.2. By substituting Equation (5) into Equation (2), the constraint-augmented measurement equation becomes

(10) yA,t−([y˙G,t×]+[yG,t×][yG,t×])dS=gZSt+aSε,t+nA.

From Equation (8) and Equation (10), the measurement vector $Z_{t}$, observation matrix $H_{t}$, and measurement noises $v_{t}$ are

(11) zt=yA,t−([y˙G,t×]+[yG,t×][yG,t×])dS,

(12) $$H_{t}=gI,$$

and

(13) vt=nA+aSε,t

As $n_{A}$ and aSε are uncorrelated, the measurement noise covariance matrix, $M_{t-1}(=E\left[v_{t-1}v_{t-1}^{T}\right])$, can be divided into two terms, as follows:

(14) $$M_{t}=\Sigma_{A}+\Sigma_{\varepsilon ,t},$$

where $\Sigma_{A}$ is the covariance matrix of the accelerometer’s measurement noise and is set to $\sigma_{A}^{2}I$, using the noise variance of the accelerometer $\sigma_{A}^{2}$. Additionally, $\Sigma_{\varepsilon ,t}$ is the covariance matrix of the constraint equation error and is defined as E[aSε,taSε,tT]. Furthermore, under the assumption that terms [dS×]n˙G and (−[dS×][yG,t×]+2[yG,t×][dS×])nG are uncorrelated, $\Sigma_{\varepsilon }$ can be written as

**Figure 2:**
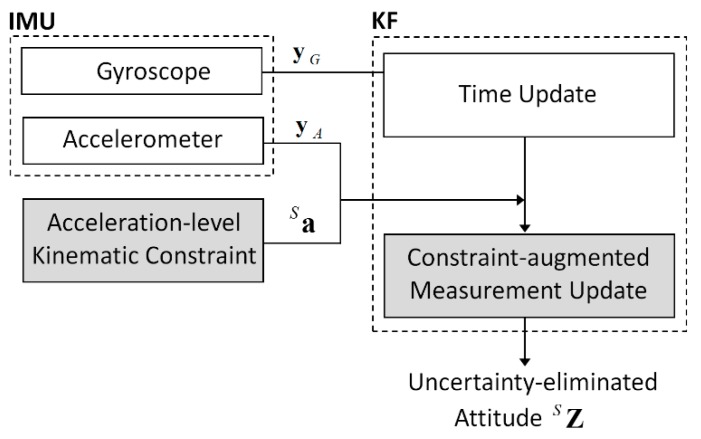
Overview of the proposed constraint-augmented Kalman filter.

(15) Σε,t=[dS×]Σn˙G[dS×]T+([dS×][yG,t×]−2[yG,t×][dS×])ΣG([dS×][yG,t×]−2[yG,t×][dS×])T

where $\Sigma_{{\dot{n}}_{G}}$ is the covariance matrix of the time derivative of the gyroscope noise and is defined as $E\left[{\dot{n}}_{G}{\dot{n}}_{G}^{T}\right]$. The covariance matrix is set to $\sigma_{{\dot{n}}_{G}}^{2}I$, where $\sigma_{{\dot{n}}_{G}}^{2}$ is the variance of the derivative of the gyroscope noise. The overall structure of the proposed KF is illustrated in Figure 2. 

## 3. Experimental Results

### 3.1. Experimental Setup and Test Conditions

For the verification of the proposed KF, which was implemented using MATLAB^®^ programming, an MPU6050 IMU sensor (from InvenSense, San Jose, CA, USA), that included a triaxial gyroscope and a triaxial accelerometer was used. The MPU6050 signals were communicated to the PC via the Arduino UNO board and entered into the proposed algorithm at a 100-Hz sampling rate (i.e., Δt = 0.01 s).

**Figure 3:**
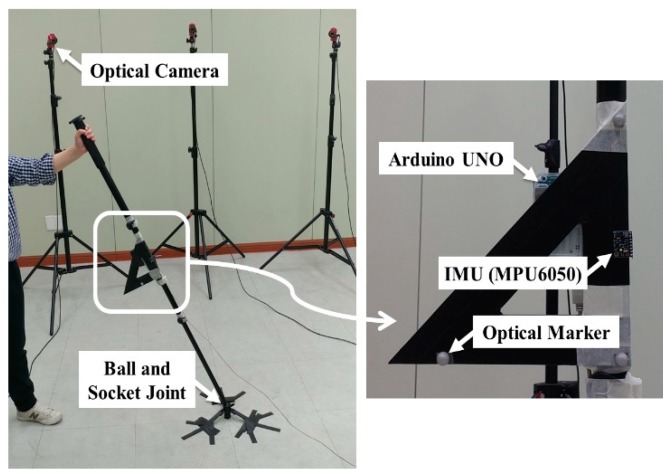
Experimental setup: An IMU and optical markers attached to a link constrained by a ball-and-socket joint.

Moreover, in order to obtain the true reference attitude, an OptiTrack Flex13 3D optical tracking system (from NaturalPoint, Inc., Corvallis, OR, USA) was used. The MPU6050 was mounted on top of a plastic right triangle ruler, and three light-emitting diode markers from the Flex 13 system were also attached to each vertex of the ruler. These markers provided a three-dimensional orientation from which the reference attitude vector ZSref could be extracted for accuracy evaluation of methods. Thereafter, the ruler was firmly fixed on a link whose end was located at the ground via a ball joint. Therefore, although the link could be rotated in any direction, its translational motion was constrained by the ball joint. The link-fixed vector from the origin of the sensor frame to the joint center was dS=[−0.451.8582.45]T cm. The link was shaken by hand in a random manner under the following conditions (see Figure 3):

- Test 1 (fast motion): Averaged ∥aS∥ of 5.96 m/s^2^ with a maximum of 23.99 m/s^2^ for a total test duration of 120 s.
- Test 2 (very fast motion): Averaged ∥aS∥ of 9.38 m/s^2^ with a maximum of 47.96 m/s^2^ for total test duration of 125 s.
- Test 3 (very fast motion for a long duration): Averaged ∥aS∥ of 9.07 m/s^2^ with a maximum of 44.44 m/s^2^ for a total test duration of 520 s.

Note that all of the three tests above were performed under highly dynamic conditions to observe the effect of constraint augmentation on the determination performance under accelerated conditions. Tests 2 and 3 had higher acceleration magnitudes than test 1. The difference between tests 2 and 3 is the test duration, i.e., test 3 had a considerably longer test duration than test 2, and accordingly, IMU was exposed to the severe condition longer.

For each of the aforementioned tests, results of the proposed KF (method A) were compared with those of the other four methods. Method B was the unconstrained KF introduced in Reference [17] and was also the platform of method A. In method B, $c_{a}$, the external acceleration model parameter that was dimensionless, was set to 0.01, which was experimentally chosen to produce satisfactory results for the three tests. Method C was another constrained KF, which adopted the estimate projection introduced by Simon et al. [30], but used the same acceleration-level constraint equation in Equation (6) formulated for the proposed method, method A. Therefore, the only difference between methods A and C was the type of constraint augmentation used. Method D was a quaternion-based unconstrained KF, presented in Reference [10] by Madgwick et al. The dimensionless-filter gain associated with gyroscope-measurement errors used in Reference [10] was set to 0.026, which was also experimentally chosen to produce satisfactory results. Method E was a direct method from measurements without using a KF, i.e., the attitude was simply determined from the equation ZS=(yA−aS)/g, where the external acceleration in Equation (6) was used after the low-pass filtering with a cutoff frequency of 10 Hz. The determination accuracy was evaluated in terms of the root-mean square-error (RMSE) of Euler angles (i.e., roll and pitch). Table 1 lists the roll and pitch RMSEs estimated from five different algorithms for the three tests.

### 3.2. Results

Regarding test 1, in the cases of methods B and D, which were unconstrained KFs, method B performed well in spite of the accelerated condition, but method D was affected by the condition, i.e., the average RMSE of the roll and pitch from method B was 2.44°, whereas that from method D was 6.50°. Both methods A and C, embedding the kinematic constraint on method B, exhibited slight improvements in comparison with method B, i.e., 1.93° from method A and 2.15° from method C. Method E, which was the direct method that used the IMU signal and constraint, yielded a higher error (3.86°) than methods A, C, and B, because of the severe noisiness of the data. 

**Figure 4:**
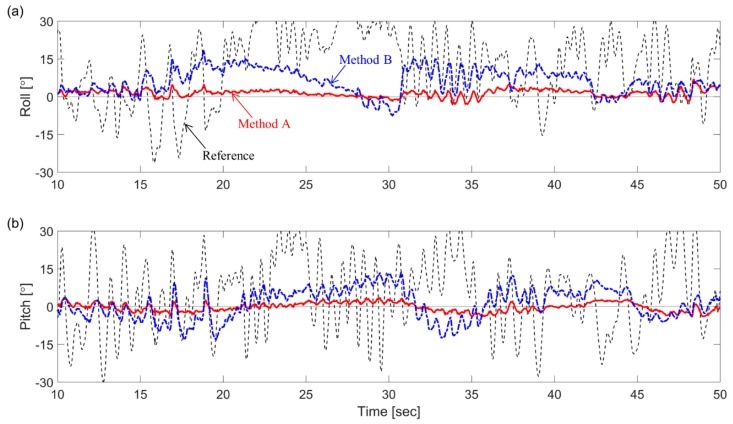
Results of Test 2: (**a**) roll and (**b**) pitch estimation errors from method A and method B during 10–50 s.

Because of the considerably fast-motion condition of test 2, methods B and D produced high values of averaged RMSEs, i.e., 5.46° from method B and 7.82° from method D. In contrast, methods A and C produced a similar level of RMSEs with test 1, in spite of the more severe condition of test 2, i.e., 1.85° from method A and 2.13° from method C. Figure 4 shows determination errors from methods A and B for 40 s (10–50 s) out of the total duration of 125 s in test 2. Because method A used the kinematic constraint, it had a relatively uniform error of less than 7° in the entire section, regardless of experimental conditions in terms of external acceleration. On the contrary, the determination error from method B highly fluctuated. Particularly, the roll determination error reached a maximum of 18.5° in 18 s. This difference can be explained by answering the question of how to deal with the external acceleration. The proposed method (method A) used the kinematic constraint equation, which was independent from the acceleration condition, whereas the unconstrained method (method B) used the stochastic Markov chain-based acceleration model, which could not cope with the rapidly changing acceleration [13].

In test 3, methods A and C were superior to the other methods, as they also were in the other tests. Because of the prolonged exposure to accelerated conditions, method D produced the largest determination error (9.11°) among the five methods. Method E performed better than method D despite of its simplicity without a KF. It should be noted that method E was free from drift because of the use of the kinematic constraint.

## 4. Discussion and Conclusions

This paper presented a novel KF, which eliminated the acceleration-related uncertainty for robust IMU-based attitude determination when IMU was attached to a constrained link. Particularly, this research dealt with an acceleration-level kinematic constraint, derived by a ball-and-socket joint. The constraint replaced the stochastic Markov chain-based acceleration model in the measurement model of the attitude determination KF. 

Our experimental results showed the superiority of the proposed KF (method A) over unconstrained KFs (methods B and D); in method A, accuracy improved by an average of 1.88° and a maximum of 4.18° in comparison with method B, and by an average of 5.85° and a maximum of 7.70°, in comparison with method D. More importantly, whereas the accuracies of conventional KFs were dependent on test acceleration conditions to some extent (that is, acceleration-related uncertainty), that of the proposed KF was completely independent of test conditions. The maximum RMSE from the proposed KF was just 2.34° of the pitch in test 3. This robustness of the attitude determination comes from the accurate estimation of the external acceleration by using the kinematic constraint (see the summary of RMSEs of the external acceleration in Table 2). 

There was no significant difference between methods A and C in terms of the determination accuracy, as both used the same constraint and KF platform. However, whereas method A used the constraint instead of the Markov-chain acceleration model, method C used the constraint after the acceleration model. In other words, method A only had one correction step using the constraint, but method C had two correction steps using the acceleration model followed by the constraint. This resulted in the difference in calculation cost, i.e., the ratio of the cost of method A to that of method C is 1.25.

Even in the proposed constrained KF, an error of approximately 2° uniformly occurred in all tests. This error level (i.e., <2.5°) was sufficient in general for the dynamic tracking of human motions and robotic systems. For example, marginal RMSE for the manual routine task was 3.65° in Reference [16]. The violation of the sensor-to-joint center position vector was expected because of the link’s bending, the fixation instability of the socket attached to the ground, and the low-cost IMU performance degradation, which were causes of error. If these causes of error are removed, the determination performance of the proposed method may be further improved. It is noticeable that the accuracies of conventional KF were dependent to some extent on acceleration conditions, whereas those of the proposed KF were independent of these conditions. Therefore, if the IMU-based attitude determination is applied for a case where the motion is restricted by a joint constraint, the accuracy and robustness of the determination may be improved by using the constraint instead of approaching the case as a general-purpose underdetermined problem. 

In this study, the socket-side link was fixed to the ground in order to simplify the problem. The proposed method can be effectively applied to the attitude determination of shanks during stance phases for pedestrian navigation systems. In addition, the method can be useful for manipulator kinematics for a link rotating around a static point. Note that the attitude provided from the proposed method is an absolute attitude with respect to the gravity and is different from the angles through joint-attached encoders. Furthermore, as a byproduct, the proposed method can determine the vertical position of the point in the segment (or the link), of which the attitude was considered in this study, for example, by calculating (ZS)TdS.

Although the direct application of the proposed method is currently limited, this paper provides a proof-of-concept for the first time, in that the uncertainty induced by the external acceleration can be removed by the augmentation of acceleration-level kinematic constraints and thus the robust attitude estimation may be possible even under highly severe operation conditions. Due to the robustness of the proposed KF, it may be applied when an accurate attitude estimation is needed regardless of dynamic conditions.

## Figures and Tables

**Table 1:** Root mean square errors of attitude (unit: °).

		Method A	Method B	Method C	Method D	Method E
Test 1	Roll	1.68	2.25	1.89	6.86	4.18
	Pitch	2.18	2.63	2.41	6.14	3.54
	Average	1.93	2.44	2.15	6.50	3.86
Test 2	Roll	2.04	6.22	2.30	8.39	5.86
	Pitch	1.65	4.70	1.96	7.25	4.08
	Average	1.85	5.46	2.13	7.82	4.97
Test 3	Roll	1.89	3.27	1.92	9.59	7.31
	Pitch	2.34	4.00	2.44	8.63	4.37
	Average	2.12	3.64	2.18	9.11	5.84

**Table 2:** Root mean square errors of external acceleration (unit: m/s^2^).

	Method A	Method B	Method C	Method D	Method E
Test 1	0.31	0.38	0.34	1.07	0.65
Test 2	0.27	0.83	0.33	1.25	0.85
Test 3	0.33	0.59	0.35	1.47	1.01
